# Cerebral microcirculatory pulse wave propagation and pulse wave amplitude mapping in retrospectively gated MRI

**DOI:** 10.1038/s41598-023-48439-0

**Published:** 2023-12-04

**Authors:** Norman Kornemann, Filip Klimeš, Agilo Luitger Kern, Lea Behrendt, Andreas Voskrebenzev, Marcel Gutberlet, Mike P. Wattjes, Frank Wacker, Jens Vogel-Claussen, Julian Glandorf

**Affiliations:** 1 Institute for Diagnostic and Interventional Radiology, Hannover Medical School, Hannover, Germany; 2 Biomedical Research in End-Stage and Obstructive Lung Disease Hannover (BREATH), Member of the German Centre for Lung Research (DZL), Hannover, Germany; 3 Institute for Diagnostic and Interventional Neuroradiology, Hannover Medical School, Hannover, Germany

**Keywords:** Medical research, Neurology

## Abstract

To analyze cerebral arteriovenous pulse propagation and to generate phase-resolved pulse amplitude maps from a fast gradient-echo sequence offering flow-related enhancement (FREE). Brain MRI was performed using a balanced steady-state free precession sequence at 3T followed by retrospective k-space gating. The time interval of the pulse wave between anterior-, middle- and posterior cerebral artery territories and the superior sagittal sinus were calculated and compared between and older and younger groups within 24 healthy volunteers. Pulse amplitude maps were generated and compared to pseudo-Continuous Arterial Spin Labeling (pCASL) MRI maps by voxel-wise Pearson correlation, Sørensen-Dice maps and in regards to signal contrast. The arteriovenous delays between all vascular territories and the superior sagittal sinus were significantly shorter in the older age group (11 individuals, ≥ 31 years) ranging between 169 ± 112 and 246 ± 299 ms versus 286 ± 244 to 419 ± 299 ms in the younger age group (13 individuals) (P ≤ 0.04). The voxel-wise pulse wave amplitude values and perfusion-weighted pCASL values correlated significantly (Pearson-r = 0.33, P < 0.01). Mean Dice overlaps of high (gray) and low (white matter) regions were 73 ± 3% and 59 ± 5%. No differences in image contrast were seen in the whole brain and the white matter, but significantly higher mean contrast of 0.73 ± 0.23% in cortical gray matter in FREE-MRI compared to 0.52 ± 0.12% in pCASL-MRI (P = 0.01). The dynamic information of flow-related enhancement allows analysis of the cerebral pulse wave propagation potentially providing information about the (micro)circulation on a regional level. However, the pulse wave amplitude reveals weaknesses in comparison to true perfusion-weighting and could rather be used to calculate a pulsatility index.

## Introduction

Age-dependent degeneration of the vascular system causes a majority of morbidity and mortality in western societies^1, 2^. In this regard, vascular stiffness is a key parameter, which increases due to fragmentation of the elastin protein in the extracellular matrix and further remodeling processes leading to arteriosclerosis. These processes increase the pulse wave transit time (PTT), amplify and accelerate pulse wave reflection, which lead to higher systolic aortal blood pressure, pulse pressure and cardiac afterload^3, 4^. Especially in organs with a high resting flow like the brain or the kidneys, the microcirculation is exposed to increasing pulse wave pressure potentially causing progressive end organ damage. This is one factor of an increasing burden of neurological diseases in aging societies^5, 6^.

The pulse wave velocity and PTT are established parameters for the vascular status potentially preceding atherosclerotic plaque formation^7, 8^ and predicting cardiovascular events^7–10^. This is related to the amount of cerebral microvascular damage^11^ and the risk for ischemic stroke or dementia^9, 12, 13^. However, the parameters are commonly acquired within arteries, e.g. the aorta or in larger arteries of organs using ultrasound, phase contrast MRI, plethysmography or invasive techniques^14–16^.

Despite the damping of the pulse pressure by the so-called “Windkesseleffekt” in large arteries^17^, it has been demonstrated that pulsatility is measurable in the parenchymal microvasculature^18–21^ as well as within the cerebral sinus^21–23^. This could indicate, that the cerebral venous pulse may not only be generated by venous compression, but that the pulse wave could also be propagated throughout the microvasculature^24–26^. In fact, a summation of several effects might cause the venous pulsatility. Calculating the arteriovenous PTT previously described as arteriovenous delay could therefore indicate age-dependent microvascular degeneration^24^. It has been suggested that a shortened delay of the pulse wave between cerebral arteries and the superior sagittal sinus may be related to normal pressure hydrocephalus^24^. However, it is still unclear which pathway dominates the conductance into the veins in healthy individuals^27^.

Using magnetic resonance imaging (MRI), the pulse wave is detectable as a superimposition of flow-related enhancement (FREE), which is already exploited for non-contrast angiography (TOF-MRA)^28^ or to generate perfusion-weighted maps based on the amplitude^29^. Flow-related enhancement occurs especially during steady-state imaging using fast gradient-echo sequences with a high number of radio frequency pulses within a short period of time. The signal decreases towards the steady-state, in which the static spins are saturated. During the steady-state, the recovery of the longitudinal magnetization is equal to the total transverse magnetization of the following radio frequency pulse so that the signal remains relatively stable^30^. During each heart cycle, fresh non-saturated spins flow into the imaging plane and lead to signal enhancement parallel to the pulse wave. Retrospective rearrangement of the images according to the heart action by postprocessing methods allows the calculation of the phases of the pulse wave together with amplitude maps offering a degree of perfusion-weighting^29^.

In this regard, it has to be emphasized, that pulse wave propagation and perfusion are two complementary physiological properties. While the pulse wave travels at speeds of several m/s, the microcirculatory perfusion occurs around mm/s. In this study, a retrospective k-space gating algorithm using the physiological monitoring unit (PMU) signal from an attached pulse oximeter is used for reconstruction of brain MR images according to the cardiac cycle to extract both complementary physiological information.

The aims of this study are firstly, to compare the pulse wave propagation in terms of arteriovenous delays of cerebral vascular territories in a healthy cohort between two age-groups.

Secondly, we aim to compare the degree of perfusion-weighting of pulse wave amplitude maps with true perfusion imaging of the reference technique pseudo-continuous Arterial Spin Labelling (pCASL)-MRI.

## Methods

### Study participants and methods

The study was approved by the Institutional Review Board, Nr. 9475_BO_K_2020, Nr. 9651_BO_S_2021 (Ethikkommission der Medizinischen Hochschule Hannover). The procedures used in this study adhere to the tenets of the Declaration of Helsinki. Consent to participate was voluntarily and written informed consent was obtained from all the participants before inclusion. Twenty-four healthy volunteers (13 males and 11 females, age range 20–61 years (IQR 24–38) with a median age of 28 years) underwent gradient echo balanced steady-state free precession (bSSFP) imaging and pCASL-MRI on a 3T scanner (MAGNETOM Vida, Siemens Healthcare, Erlangen, Germany) using a 16-channel head coil in head-first supine position. Two age cohorts were compared, an older cohort ≥ 30 years with 5 females and 6 males with a median age of 46 years (IQR 31–49 years) and a younger cohort ≤ 29 years with 6 females and 7 males with a median age of 25 years (IQR 23–27 years).

### Imaging parameters

For both bSSFP and pCASL sequences, two axial slices were acquired at the center of the corpus callosum (slice 1) and 3 cm above (slice 2) to generate a larger sample of slices with differing anatomic structures with and without ventricles, basal ganglia with perforating arteries.

The following settings of the bSSFP sequence were used for FREE-MRI: matrix size 128 × 128 (interpolated to 256 × 256 via bicubic interpolation), field of view (FOV) 340 × 340 mm^2^, slice thickness 5 mm, bandwidth 1500 Hz/px, repetition time (TR) 3.6 ms, echo time (TE) 1.6 ms, flip angle 60°, 500 repetitions, total acquisition time of 3.84 min per slice. The sequence was not fully balanced, as TR was minimized to accelerate imaging. A pulse oximeter was attached to the index finger of each study participant in order to record the PMU signal. The oximeter was synchronized with the data acquisition to ensure that each readout has a specific timestamp related to the pulse trigger from the pulse oximeter.

The parameters of the pCASL with matching resolution and FOV were used according to Alsop et al.^31^: matrix size 128 × 128 (interpolated to 256 × 256), FOV 340 × 340 mm^2^, slice thickness 5 mm, bandwidth 1776 Hz/px, TR 4900 ms, TE 25 ms, echo spacing 0.61 ms, flip angle 90°, 45 measurements, labeling duration 1800 ms, post labeling delay 1800 ms, total acquisition time of 3.7 min per slice.

### Reconstruction across the cardiac cycle

**Figure 1:**
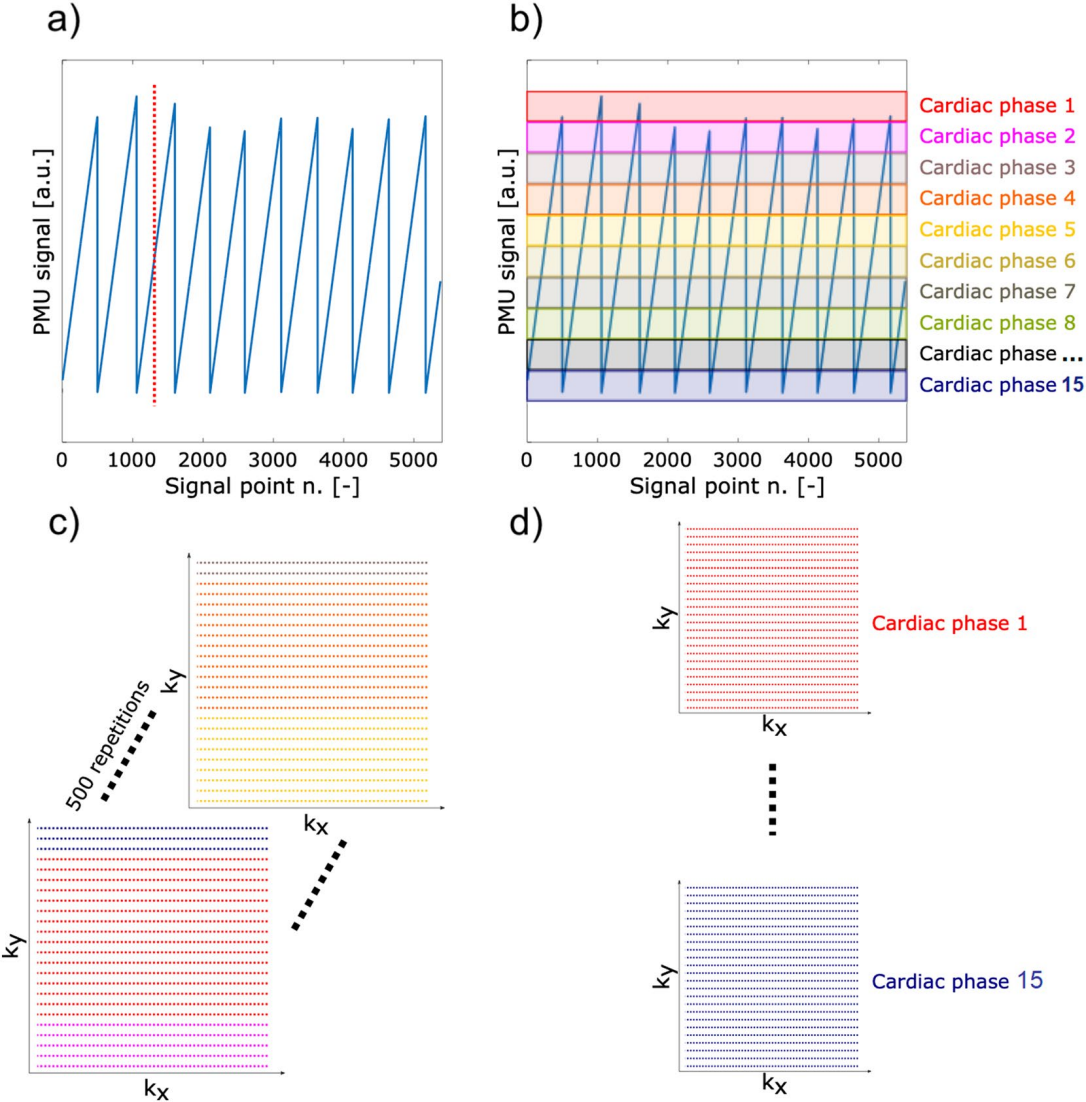
Postprocessing scheme of FREE-MRI. In (**a**) a PMU signal is depicted with its respective steady-state cut highlighted by the red dotted line. The PMU timestamp signals the start of each heart cycle indicated by the vertical lines. Afterwards, the steadily increasing PMU signal is binned into 15 cardiac phases using a gating window (**b**). (**c**) Represents the unsorted k-space data (10 out of 500 repetitions were discarded to fulfill steady-state signal condition) acquired using bSFFP sequence with cartesian sampling. Each imaging readout (marked as dotted lines) has its corresponding PMU signal, which is assigned to a related cardiac phase (different colors) (**d**). All k-space data with a cardiac phase within each window in (**b**) are accepted to reconstruct representative cardiac phases. After averaging of k-space lines with same encoding in the same cardiac phase, 15 images representing 15 cardiac phases are reconstructed.

An example of the PMU timestamps describing the time from the last gating signal by the pulse oximeter is shown in Fig. 1a. The obtained PMU signal is modulated by cardiac action, where the maxima correspond to the end-systole. In order to ensure that the magnetization reached its steady-state, the first 10 repetitions with 1280 acquired imaging readouts and respective 1280 PMU signal points were discarded (Fig. 1a, red dotted line). Next, a peak detection of the PMU signal was performed using a local threshold-based algorithm. Afterwards, the PMU signal was binned into subintervals representing 15 cardiac phases by a gating window with same width as illustrated in Fig. 1b.

Each acquired k-space line was sorted according to its position within the cardiac cycle into one of the cardiac phases (Fig. 1c). This means, that a slightly different number of k-space lines with the same encoding could be present in each cardiac phase. In theory, each phase encoding step should have about 33 (500/15) k-space lines. To guarantee a negligible influence by the small varying number of sorted k-space lines in each encoding step, the multiple k-space lines within each specific cardiac phase were averaged prior to image reconstruction (Fig. 1d). Finally, a complete cardiac cycle containing 15 cardiac phase images was created. Inverse discrete Fourier transform was used for the reconstruction of each receive channel image which were combined using the sum of squares approach. In order to enhance the signal-to-noise (SNR) of the reconstructed images, an image-guided filtering was applied on the whole cardiac cycle images^32^.

### Pulse wave phase maps

**Figure 2:**
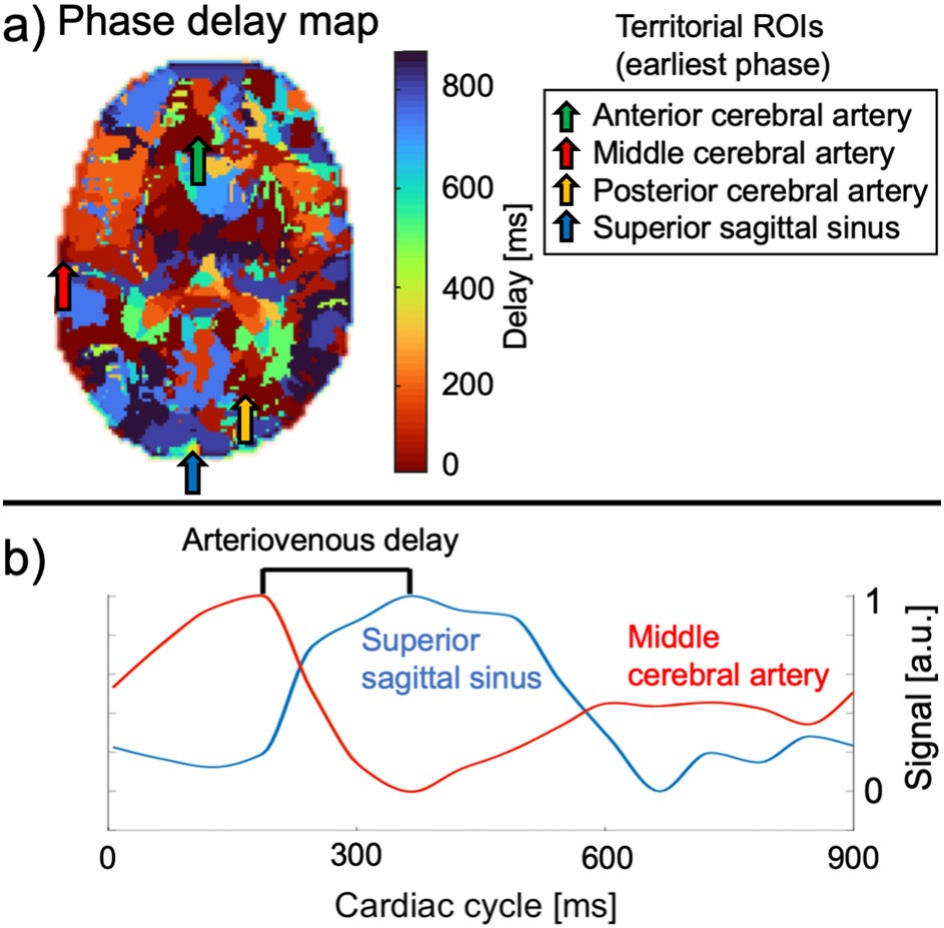
Phase map and calculation of arteriovenous delay. In (**a**), the phase map depicts various phases of the pulse wave across the vascular territories. The arrows show the earliest phases in each vascular territory and the superior sagittal sinus. In (**b**), normalized signal waveforms of voxels in the territory of the middle cerebral artery and the superior sagittal sinus are shown to calculate the arteriovenous delay as the phase difference between both maxima.

After reconstruction, there are 15 images of different heart phases and each voxel has a variable signal across the phases. While the phase with the maximum average signal of the entire brain might be in a certain part of the heart cycle, the maximum signal of each individual voxel can be in a differing phase. Phase maps were then generated by calculating the difference of the phases in which each individual voxel and the entire brain display their maximum signal. As there are 15 reconstructed phases, there is also a limited amount of 15 individually labelled phase differences resulting in the patchy appearance of the map. Assuming a heart rate of 1 beat per second, each heart phase would be 67 ms apart (1 s/15 phases). An exemplary phase map is presented in Fig. 2a. Regional arteriovenous delays of the pulse wave were calculated by subtracting the phase delays between regions with the least phase delay in the anterior, middle and posterior cerebral artery territories (ACA, MCA and PCA) and the superior sagittal sinus (Fig. 2b). Measurements were performed for both sides at the level of the center of the corpus callosum in which the vessels were constantly depicted.

### Pulse wave amplitude maps

**Figure 3:**
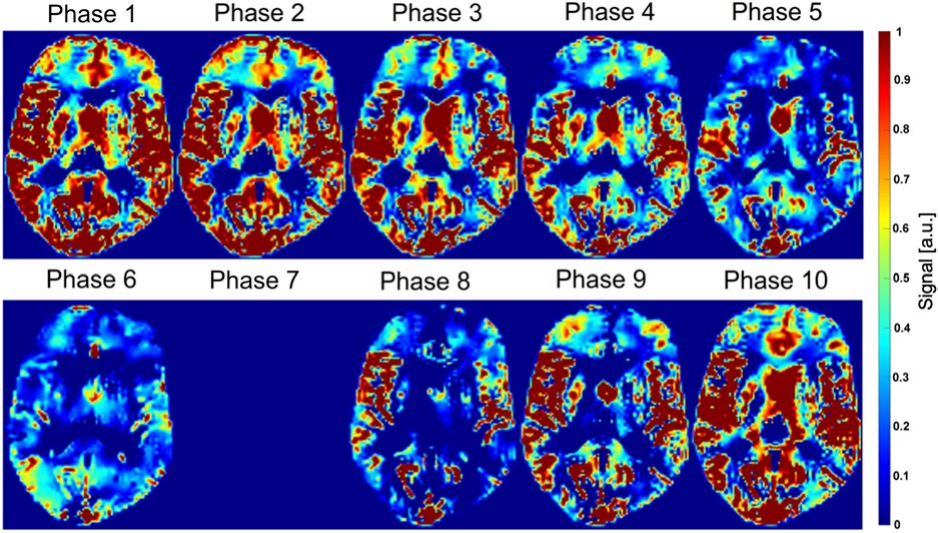
Pulse wave amplitude maps. Pulse wave amplitude maps of each cardiac phase can be generated by subtractions of the minimum phase (in this case phase 7) from each phase (Phase n − Phase min). Notice a subtraction of the minimum phase from itself results in a zero map (Phase 7).

The amplitude of the signal change across the heart cycle caused by the flow-related enhancement was then calculated. The amplitude of each phase was calculated by the signal difference of each heart phase towards the phase with the lowest average signal of the entire the brain parenchyma^29^ (Fig. 3).

**Figure 4:**
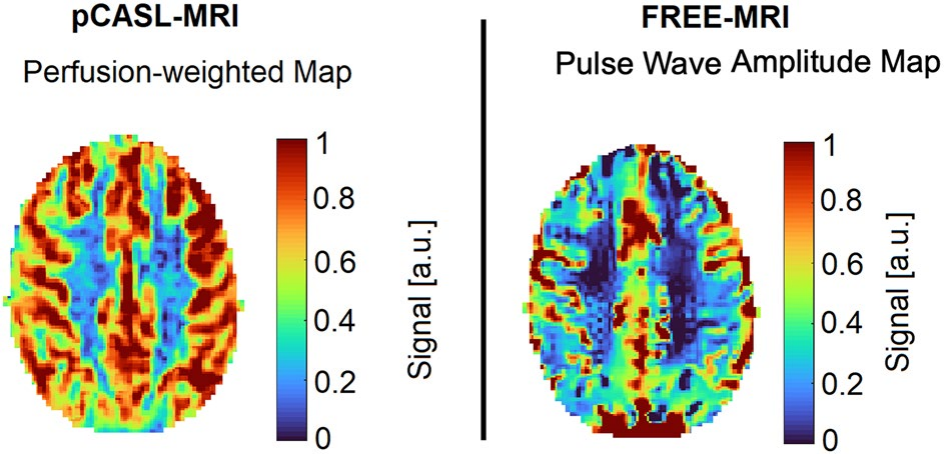
Exemplary results of pCASL-MRI and FREE-MRI. Perfusion-weighted pCASL map (on the left) and flow-related enhancement (FREE) pulse wave amplitude map (on the right). Whereas pCASL-maps are based on the difference between labelled- and control-images, the amplitude of the pulse wave in the maximum signal phase is mapped to obtain the amplitude map in FREE-MRI. To allow a qualitative comparison, both maps were normalized to the 90th percentile of its values with a maximum value of 1.

Perfusion-weighted pCASL-maps were generated by averaging the subtractions of control and label images (Fig. 4).

The signal contrast was calculated by dividing the mean amplitude value or the signal change between labelled and contol images by the mean image signal and measures the changing signal proportion for each technique^33, 34^. For pCASL-MRI, the mean signal was calculated by the average signal of the tagged and control images together. For FREE-MRI, the mean signal was calculated as the average signal of all 490 reconstructed images (500 images minus 10 images until the steady state was reached).

### Spatial overlap between FREE-MRI and pCASL

**Figure 5:**
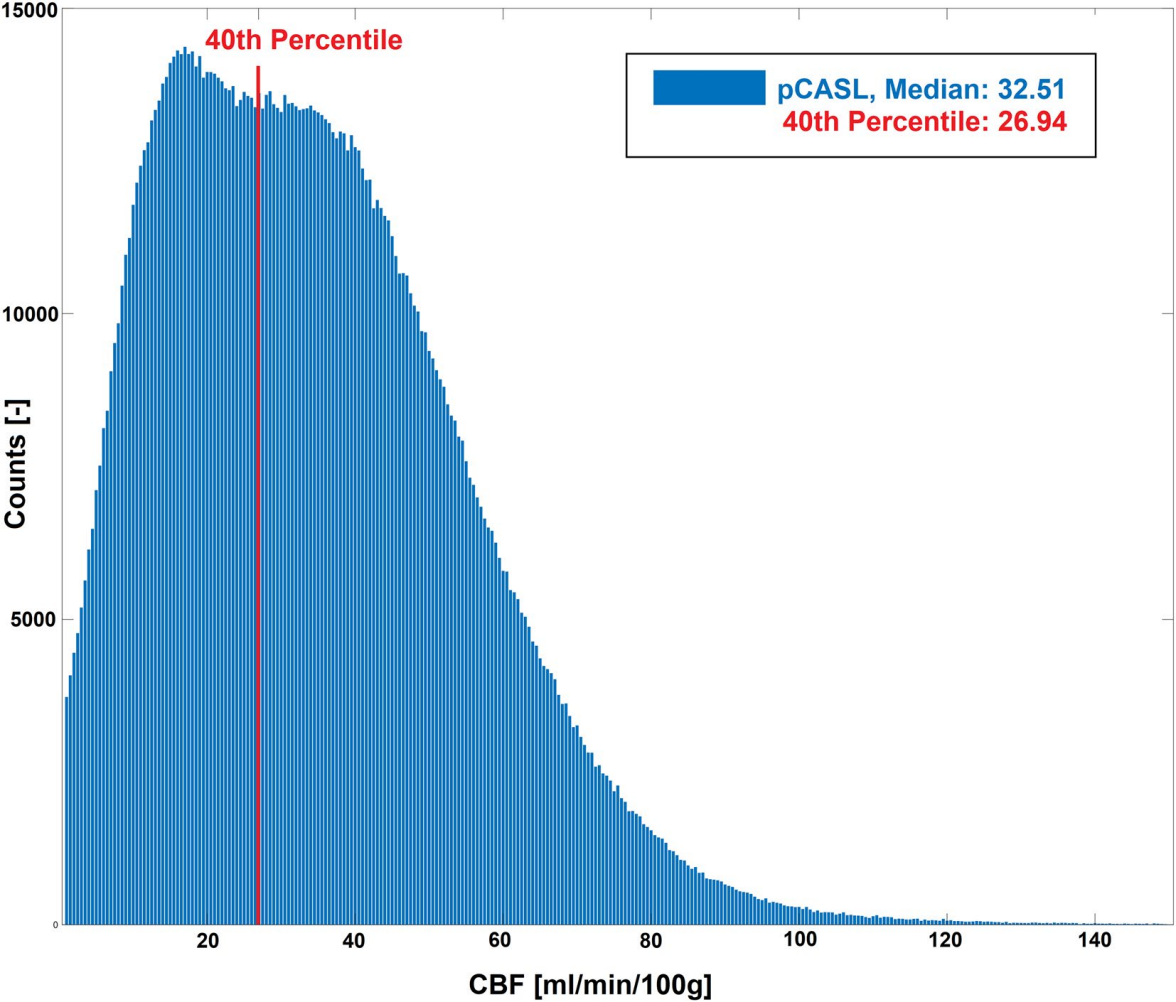
A histogram of all pCASL-values of all subjects is indicating a separation of white (lower peak) and gray (higher peak) matter voxels around the 40th percentile. Quantification of the cerebral blood flow (CBF) was performed using the BASIL toolbox v.6.0.6.4^36^ from the FSL library of the Analysis Group, FMRIB, Oxford, UK^37–39^. Voxel wise calibration was achieved with a proton density M0 image. A well-mixed exchange model with no dispersion, a T1 value of 1.65 s for blood, an inversion efficiency of 0.85, a fixed label duration and an adaptive spatial regularization on perfusion was chosen according to the white paper mode^31^.

**Figure 6:**
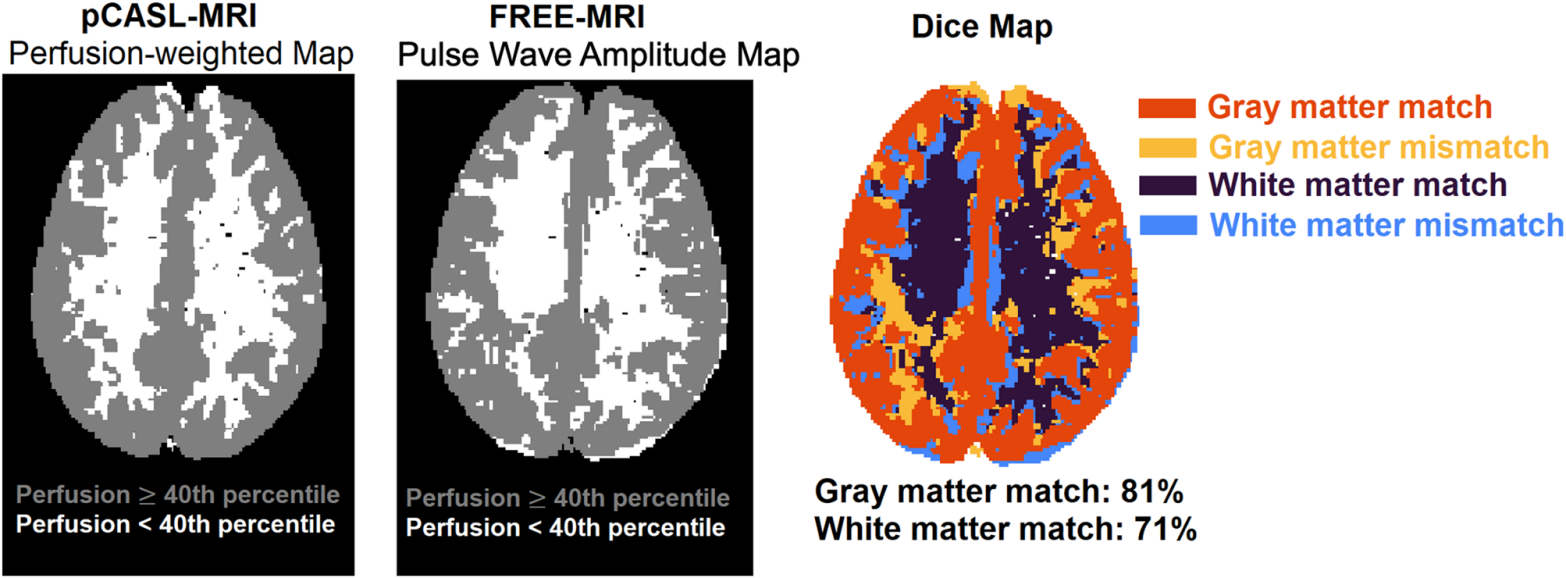
Binary maps were generated using the 40th percentile of the values as a threshold. The values of both techniques were the signal changes between labelled and unlabelled images or the signal changes due to the amplitude, respectively. Voxels above were considered as highly perfused (gray matter) and below as lowly perfused (white matter) voxels. The binary maps were compared using a Dice map showing the overlap and mismatch at the interface of gray and white matter between both techniques.

For the spatial overlap analysis, the FREE-MRI pulse wave amplitude maps and the pCASL-MRI perfusion-weighted maps of each study participant were converted into binary maps using the 40th percentile of the values as a threshold according to the average volume fraction of gray and white matter in healthy individuals^35^. This ratio is maintained as both compartments have significantly differing perfusion characteristics and are commonly separated in histograms (Fig. 5). Thus, voxels having less than this threshold should represent white matter and vice versa. Any deviation from this assumption reveals spatial differences between both techniques and can be quantified using the Sørensen-Dice coefficient as illustrated in Fig. 6.

### Software and statistics

MATLAB R2020b (The MathWorks, Natick, MA, USA) was used for postprocessing and analysis. All data and code is available in de-identified form the corresponding author upon request with a formal data and/or code sharing agreement. Differences between FREE-MRI derived values and pCASL-MRI values were evaluated using a Wilcoxon-signed rank test considering the non-normality distribution. Arteriovenous delays were compared in regions of interest between younger and older subjects using a Mann–Whitney-U-Test. Voxel-wise correlation between both maps was performed with the Pearson correlation. The resulting Pearson correlation coefficients were tested for significance using Fisher’s Z transformation. The spatial overlap of the binary maps was determined with Sørensen-Dice coefficients. P-values ≤ 0.05 were considered as significant.

## Results

### Pulse wave propagation: arteriovenous delays of young vs. old cohort

For the evaluation of the arteriovenous delays, the cohort was separated into a younger cohort with 13 individuals being 29 years or younger with a median age of 25 years and into an older cohort with 11 individuals being 30 years or older with a median age of 46 years.

**Figure 7:**
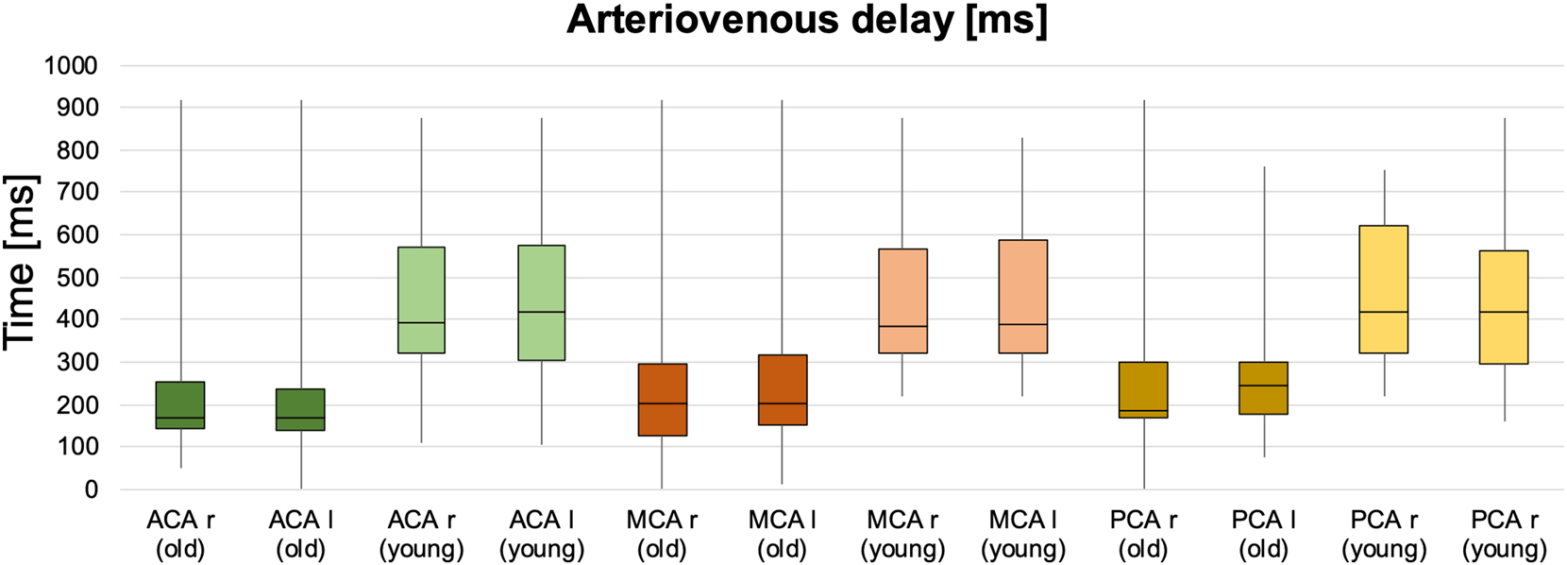
The comparison was done in the territories of the anterior, middle and the posterior cerebral arteries (ACA, MCA and PCA) for both sides (r and l). All locations revealed significant differences between both age groups with *P*-values ≤ 0.04. Young cohort in lighter colors.

The arteriovenous delays between the earliest phase in each vascular territory and the superior sagittal sinus were significantly shorter in the older age group across all vascular territories (*P* ≤ 0.04) (Table 1 and Fig. 7).

**Table 1:** Comparison of median arteriovenous delay values in milliseconds ± IQR between both age groups in anterior, middle and the posterior cerebral arteries (ACA, MCA and PCA) for both right and left hemispheres (r and l).

Arteriovenous delay in territory [ms]	Old group	Young group	Mann–Whitney-U-Test
ACA r	169 ± 112	394 ± 249	*P* = 0.02*
ACA l	169 ± 112	419 ± 269	*P* = 0.02*
MCA r	204 ± 169	286 ± 244	*P* = 0.02*
MCA l	204 ± 164	289 ± 266	*P* = 0.02*
PCA r	185 ± 125	419 ± 299	*P* = 0.02*
PCA l	246 ± 299	419 ± 267	*P* = 0.04*

The arteriovenous delay is the time difference between the peak signal of the earliest phase in each vascular territory and the superior sagittal sinus. *P*-values marked with * are significant.

### Perfusion-weighting: pulse wave amplitude maps vs. pCASL-MRI

The voxel-wise Pearson correlation between pulse wave amplitude maps of FREE-MRI and perfusion-weighted maps of pCASL-MRI were highly significant with a mean correlation coefficient of 0.33 ± 0.09 (*P* < 0.01).

The spatial overlap of the binary maps resulted in an average Dice coefficient of 73 ± 3% and of 59 ± 5% for the gray matter and the white matter, respectively.

The analyses of the signal contrast showed similar contrast for FREE-MRI and pCASL-MRI within the whole brain and white matter and significantly higher values for FREE-MRI within the gray matter (P < 0.01*) (Table 2).

**Table 2:** Signal contrast of FREE and pCASL-MRI.

Signal contrast [%]	FREE	pCASL	Wilcoxon-signed rank test
Whole brain	0.50 ± 0.13	0.42 ± 0.10	*P* = 0.21
Grey matter	0.73 ± 0.23	0.52 ± 0.12	*P* < 0.01*
White matter	0.32 ± 0.07	0.29 ± 0.08	*P* = 0.42

The values are expressed as mean ± standard deviation. Values with * show a significant *P*-value.

## Discussion

In this study, we presented the feasibility to exploit flow-related enhancement to analyze the pulse wave propagation within the cerebral vascular territories. On one hand, this enables the analysis of the pulse wave propagation via the calculation of the arteriovenous delay on a regional level, and on the other hand, a weak degree of perfusion-weighting is achieved by mapping the signal amplitude. This combines two complementary physiological features that have been acquired only by separate technical approaches before.

Considering the analysis of the cerebral pulse wave propagation, this has previously been determined within larger vessels using phase contrast MRI or sonography^23, 24^. In our study, we were able to display different phases within each vascular territory representing different distances towards the microvasculature. When comparing regions with the earliest phases within each vascular territory—considered as larger arteries –, our results are in line with previous reports calculating the cerebral arteriovenous delay using phase contrast MRI^24^. Interestingly, similarly to measurements outside the CNS^40^, we were able to acquire substantial differences between both age groups in our healthy cohort indicating sensitivity to age-dependent vascular stiffening. Considerably shorter arteriovenous delays were calculated in the older group despite the progressive elongation of the cerebral arteries^41^ softening of the brain tissue^42, 43^ and increasing extra-axial cerebrospinal fluid (CSF) space due to progressive shrinkage of the brain^44–46^. Assuming pulse wave transmission pathways through the brain tissue and the CSF by squeezing veins, a prolonged arteriovenous delay would be expected under these circumstances in the older cohort^24^. Therefore, the shortening of the arteriovenous delay in the older cohort leads to the assumption that in healthy individuals the pulse wave is predominantly propagated through the (micro)vasculature where its speed is mainly determined by the vascular stiffness. This gives us confidence to consider the arteriovenous delay as the commonly known “pulse wave transit time” which enables a regional analysis of the cerebral vascular status with this approach. Also, a summation of several transmission pathways could cause the venous pulsations. Interestingly, no significant differences of the pulse wave amplitude were detected between both age groups revealing less sensitivity for age dependent remodeling processes. Under normal circumstances, the CSF reduces the pulse pressure by absorbing energy prior to the microvasculature^47^. However, in patients with normal pressure hydrocephalus or with increased intracerebral pressure, the pulse wave might predominantly be propagated across the CSF and the parenchyma indicating reduced compliance^48–50^.

Asymmetries of the phase maps may be due to differing anatomic orientation of both hemispheres. Some differences may also be caused by imperfect sequence planning, inaccuracies coming from binning of the PMU signal, and definition of the starting point for phase delay calculation. The length of cardiac cycle is individual for each patient depending on their cardiac rate. The entire cardiac cycle is divided into 15 phases with effective temporal resolution ranging from 8 to 133 ms assuming heart rates of 50–120 per minute in our healthy volunteer cohort. Due to the limited temporal resolution, some of the evaluated voxel delays might not be correctly calculated. For example, if small negative phase delay is between two neighboring voxels, an artificially high phase shift of almost whole length of cardiac cycle would be calculated for this voxel (e.g., 800 ms).

Considering the perfusion-weighting aspect, there are obvious differences between both evaluated techniques. Flow-related enhancement is confined to a pulsatility, which occurs predominantly in the vasculature, but also within the CSF. This is dependent on orientation of image acquisition, the amount and size of the vessels, blood volume, the flow-velocity and its turbulence. Most of the contrast in the large arteries will be related to blood that is destined to perfuse tissue in a different voxel (potentially outside of the imaging slice) and will therefore not reflect local perfusion. Additionally, as inflowing blood travels further down the vasculature, the signal will be gradually attenuated by T2-relaxation reducing its contrast compared to upstream blood, thus down-weighting the contribution of local perfusion to the contrast. The images will also be sensitive to other effects, that vary with the cardiac cycle such as CSF flow. Indeed, there appears to be FREE-MRI contrast present in the ventricles in Fig. 4.

In this regard, pCASL-MRI better reflects tissue perfusion because exclusively the inflowing arterial blood is labelled and a post-labelling delay is then used to give this labelled blood time to reach the parenchyma and leave the large arteries^51^. While the FREE-MRI images do bear some qualitative resemblance to the pCASL-subtraction images, this is to be expected because low resolution cerebral blood volume maps do look similar to low resolution CBF maps^52^. However, both techniques share signal variations due to the inflow of spins from the vasculature into the imaging plane using subtractions as their underlying mechanism for the generation of the perfusion-weighted maps. Furthermore, our results show a decent similarity indicating a weak degree of perfusion-weighting by flow-related enhancement which might be sensitive to pathologies^53^. The real value of pulse-wave amplitude maps in pathologies must be confirmed in further clinical studies.

The signal contrast of both techniques is ranging within a similar magnitude, which could indicate, that flow-related enhancement is detected quite close to the microvasculature. The substantially higher signal contrast of FREE-MRI within gray matter could be due to the high sensitivity and overrepresentation of vascular flow (arterial and venous) and partial volume effects of CSF flow. However, a higher signal contrast does not mean a more accurate depiction of perfusion. Notably, calculating the signal contrast by dividing the signal amplitude by the mean signal is very similar to the pulsatility index determined via ultrasound, calculated as (peak systolic velocity − minimum diastolic velocity)/mean velocity^54^. The pulsatility index and the similar resistance index are established parameters to assess the vascular resistance with a broad range of clinical and scientific applications, e.g., indicating coronary bypass graft dysfunction, renal dysfunction or increased intracranial pressure^55–57^. This hints towards many clinical applications for FREE-MRI without being limited to larger vessels, but with the ability to detect regional pulsatility within the parenchyma of the organ.

Regarding the spatial comparison of both techniques using the Sorensen-DICE coefficients, it must be stated, that using a single threshold to separate gray and white matter voxels creates numerous falsely labelled voxels. Despite the fact, that both compartments can be identified in Fig. 5, both compartments are overlapping and create a rather broad zone due to voxels partially containing white- and gray-matter and other tissues. Therefore, the DICE-maps should be interpreted cautiously and are intended to test the separation of gray- and white matter voxels by FREE-MRI and indicate mayor deviations (vessels in white matter or CSF flow). A further limitation of this study is the cohort with exclusively healthy volunteers. Also, in contrast to pCASL-MRI, neither a 3D-technique has been developed, nor perfusion/flow quantification have been implemented yet. Another limitation is, that while bSSFP will increase the SNR, a spoiled readout would reduce the contamination from arterial and venous blood flowing through the slice. Spins that have moved out of slice continue to contribute a considerable amount of signal with bSSFP readouts due to the balanced gradients, effectively increasing the slice width in a velocity and off-resonance dependent manner^30^. A further reduction of the slice thickness or the use of a spoiled gradient echo sequence could be pursued to reduce this.

In future studies, the influence of various factors like the slice thickness determining partial volume effects on FREE-MRI derived parameters might be examined similarly to pCASL-MRI^58^. The confounding caused by this circumstance might be relevant. Furthermore, potentials to increase imaging speed must be evaluated to cover the entire brain in a similar amount of time as in pCASL-MRI. In this regard, 500 images for one slice generates much redundancy in data, which unnecessarily increases scan time. In a future study, a step-wise reduction of the data should be performed to find a more efficient number of images per slice, while maintaining the image parameters at a similar level.

## Conclusion

The dynamic information of flow-related enhancement allows analysis of the cerebral pulse wave propagation potentially providing information about the (micro)circulation on a regional level. However, the pulse wave amplitude reveals weaknesses in comparison to true perfusion-weighting and could rather be used to calculate a pulsatility index.

## Data Availability

All data was acquired at Hannover Medical School, Germany, and is available in de-identified form from the corresponding author upon request. A formal data sharing agreement is needed. The MATLAB code is available from the corresponding author upon request. A formal code sharing agreement is needed.
